# Multimodal discrimination of immune cells using a combination of Raman spectroscopy and digital holographic microscopy

**DOI:** 10.1038/srep43631

**Published:** 2017-03-03

**Authors:** Naomi McReynolds, Fiona G. M. Cooke, Mingzhou Chen, Simon J. Powis, Kishan Dholakia

**Affiliations:** 1 SUPA, School of Physics and Astronomy, University of St Andrews, Fife, KY16 9SS, United Kingdom; 2 School of Medicine, University of St Andrews, Fife, KY16 9TF, United Kingdom

## Abstract

The ability to identify and characterise individual cells of the immune system under label-free conditions would be a significant advantage in biomedical and clinical studies where untouched and unmodified cells are required. We present a multi-modal system capable of simultaneously acquiring both single point Raman spectra and digital holographic images of single cells. We use this combined approach to identify and discriminate between immune cell populations CD4+ T cells, B cells and monocytes. We investigate several approaches to interpret the phase images including signal intensity histograms and texture analysis. Both modalities are independently able to discriminate between cell subsets and dual-modality may therefore be used a means for validation. We demonstrate here sensitivities achieved in the range of 86.8% to 100%, and specificities in the range of 85.4% to 100%. Additionally each modality provides information not available from the other providing both a molecular and a morphological signature of each cell.

Optical techniques are widely recognised for their ability to study biological systems and are often used in single cell studies. Label free techniques in particular are becoming more important, owing to the fact they do not require the addition of exogenous agents, which may interfere with biological processes, allowing studies of cells in an environment that more closely reflects their natural surroundings.

This search for powerful optical label free techniques has brought Raman spectroscopy (RS) to the fore. Raman spectroscopy provides specific molecular information of a sample by inelastic scattering of light that results in a spectrum indicative of the constituent molecular contents of a sample. RS has been used for analysis of biological cells^1^, including immune cells^2^,^3^,^4^,^5^. For each cell type the Raman spectrum can provide intrinsic information such as DNA, lipid, or protein content^6^. RS offers high specificity and has the added advantage that it does not require external tags so that we can study label-free, untouched, live cells and tissue. Whilst RS is capable of providing molecular information for the discrimination between cell types, there is no morphological information provided. Furthermore due to its small cross-section, RS is often hampered by its long acquisition times. RS has thus been a prime candidate for use along-side complimentary optical techniques. In particular an advantage would be gained by combining RS with a morphological approach such as optical coherence tomography (OCT) or quantitative phase imaging. The development of multi-modal systems for diagnostics is one of the main challenges facing biophotonics today. By combining complimentary techniques we may overcome limitations specific to a single technique and gain a more complete description of our sample. Studies combining RS with OCT have enabled the characterisation of tissue^7^ or cancers^8^,^9^ where both micro-structural and morphological information from OCT and biochemical information from RS can be jointly evaluated to provide a more complete description with future applications in assisted biopsy guidance^10^.

Shape and optical thickness are also useful parameters, particularly for the discrimination between cells, and may be recorded via quantitative phase imaging. Digital holographic microscopy (DHM), an interferometric imaging method, can provide quantitative information on the phase shifts induced by a sample^11^,^12^. DHM has proven useful for many applications such as discrimination between the maturity levels of red blood cells^13^, label-free cell counting^14^, and determining morphological information of cells for identification and disease diagnosis^15^,^16^. Furthermore DHM has rapid acquisition times capable of quantitatively studying cellular dynamics in real-time^17^.

It has been demonstrated that DHM and RS may be implemented simultaneously for determination of both local molecular content and observation of dynamic sample morphology at video rates^18^, and for determining the relationship between Raman information and quantitative phase information of a cell^19^,^20^. This technique has also been applied to red blood cells^21^ where wide field DHM imaging is used as a screening tool to look for morphological features that may indicate malaria infection, and Raman microscopy is used for validation.

The two techniques are complimentary by nature; DHM relies on the linear elastic scattering of a wave front passing through the sample, and Raman spectroscopy on the inelastic vibrational scattering from the sample. The combination of these two signatures can therefore provide a more complete description of the sample which may be of interest for applications studying cellular behaviour in a label free manner. In practical terms assembling a DHM system is relatively simple and can easily be integrated around a Raman microscope. DHM employs a narrow linewidth source, in our case implemented with an incident wavelength of 532 nm, whereas Raman excitation is performed at 785 nm, with the Raman emission covering a broad range of higher wavelengths; this makes it easy to isolate the two signals from each other, ensuring simultaneous measurements are possible. Dual modality may enable high throughput measurements in the future, where DHM may provide a fast initial screening, limited only by camera acquisition rates (up to 20 fps in live mode)^22^,^23^, and Raman spectroscopy can provide specific molecular information from cells of interest. Finally neither Raman spectroscopy nor DHM require any external tags or sample processing before measurements allowing all data to be taken in a label-free manner.

In this paper we investigate a multi-modal all-optical label-free approach for the identification of immune cells. In particular we focus on immune cell types which pose a particular challenge; in the bloodstream lymphocytes of both B and T lineages are similar in size and shape, and are also similar to natural killer (NK) and monocytes. However, the number of each of these cell types present alters significantly when the immune system is challenged during periods of infection or inflammation. Thus rapid label free analysis of numbers and cell subtypes could be of significant assistance to understand such conditions and ultimately pave the way for clinical use.

We have previously demonstrated that wavelength modulated Raman spectroscopy (WMRS) is capable of discriminating between immune cell populations CD4+ T cells, CD8+ T cells, and Natural Killer cells^4^. The main challenge we aim to overcome is the long acquisition time required to obtain Raman spectra as faster throughput rates are necessary to make this technology clinically relevant. Here, we present a multi-modal system combining RS and DHM for the characterisation and identification of immune cell populations CD4+ T cells, B cells and monocytes. This is the first time RS and DHM have ever been applied in combination to this burgeoning subject. The experimental system we have developed is capable of simultaneously recording DHM images and acquiring single point Raman spectra of single live cells. The resulting DHM phase maps are analysed to investigate the most accurate way of describing the cells for discrimination purposes. Illustrated here are the use of signal intensity histograms^24^ and texture analysis^25^,^26^,^27^ as a method to describe the phase maps. A multivariate statistical approach in the form of principal component analysis (PCA) is used for discrimination between cell types. Leave-one-out cross-validation (LOOCV) statistics are used to estimate the efficiency of each technique.

## Results

### Raman spectroscopy

**Figure 1:**
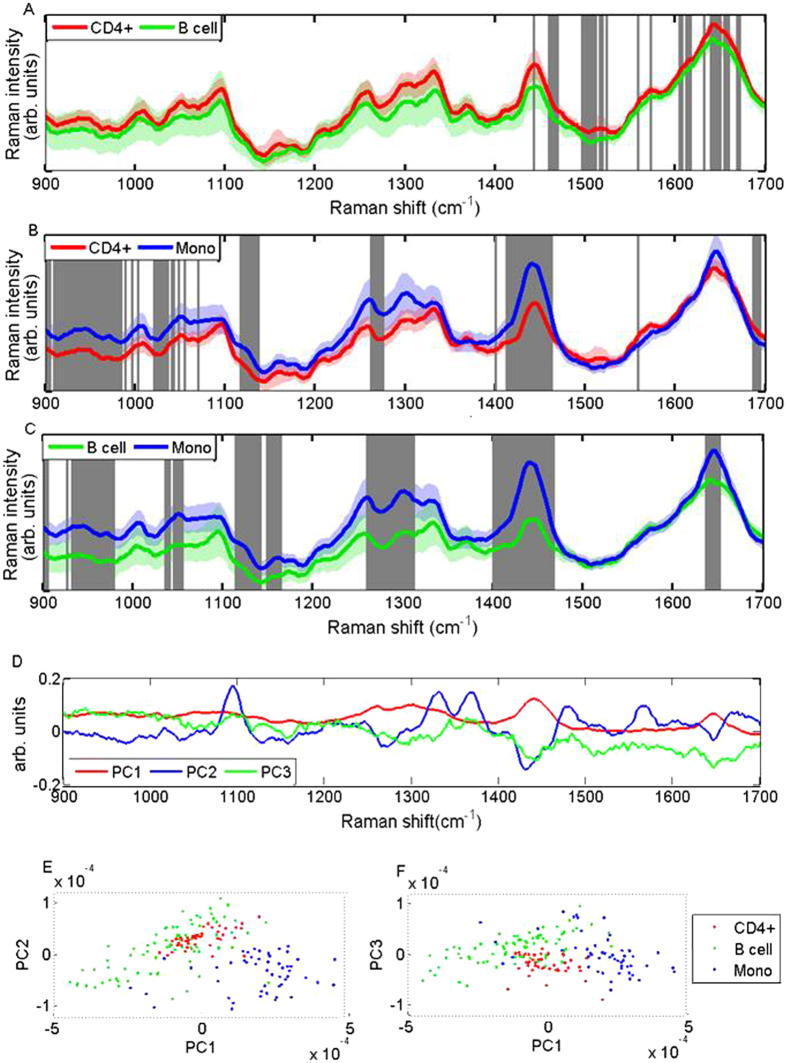
(**A**–**C**) Standard Raman spectra collected on the multi-modal system illustrating pairwise comparisons between CD4+ T cells, B cells and monocytes. Solid lines show the mean spectrum for each cell subset and shadowed regions represent one standard deviation. Grey vertical bars indicate the regions of most significant difference between cells, as calculated by the student’s t-test at a significance level of (**A**) p < 10^−8^, (**B**) p < 10^−13^, and (**C**) p < 10^−18^. (**D**) illustrates the loadings for the first 3 PCs from all cell types. The 1st, 2nd, and 3rd PCs are shown in red, blue and green respectively. (**E,F**) demonstrate scatter plots for the whole data set using the first 3 PCs. Distinct clusters for each cell subset demonstrate the ability to discriminate between cell types using RS.

Standard Raman spectra were acquired for CD4+ T cells, B cells, and monocytes; the mean spectrum for each cell type can be seen in Fig. 1(A–C). Regions of significant difference between pairwise mean spectra are highlighted according to a student’s t-test; the significance level for each pair of cell lines varies so as to best highlight the peaks of most significant difference between them. The larger the significance level required to highlight the main peaks can be interpreted as signifying that the cell lines are most similar to each other. For example comparing the relatively similar B cells and T cells requires a significance level of p < 10^−8^, whereas comparing monocytes and B cells, which are chemically most different to each other, requires a significance level of p < 10^−18^. Using these highlighted peaks we can have some knowledge of how molecular composition varies between cell populations. Raman bands of significant difference between CD4+ T cells and B cells may be identified as CH2 deformation in lipids (1455 cm^−1^), adenine/guanine (1585 cm^−1^), and amide I (1665 cm^−1^). Regions of significant difference between CD4+ T cells and monocytes include protein *α* helix and protein C-C skeletal modes (938 cm^−1^), skeletal C-C stretch in lipids (1129 cm^−1^) and CH2 adenine and guanine (1421 cm^−1^). Additional regions between monocytes and B cells include amide III (1259 cm^−1^), and adenine/amide III (1304 cm^−1^)^28^,^29^,^30^. Smith *et al*. have recently completed a study on Raman spectra of B and T cells, performed with an incident wavelength of 532 nm using a similar power and acquisition time as our study. The Raman peaks observed correlate well with our study, where they have also noted Raman bands around 1455 cm^−1^, 1585 cm^−1^ and 1665 cm^−1^ for T and B cells; their complete profile of band assignments may be found in ref. 5.

PCA analysis was performed on the whole data set using the first 10 PCs, which accounted for 94.4% of the total variance. Data points were plotted on a PC scatter plot and distinct clusters were formed for each cell population (Fig. 1D,E). Using LOOCV we derived a confusion matrix for the three cell subsets (Table 1) and pairwise sensitivities and specificities were estimated, as shown in Table 2. The average sensitivity and specificity achieved were 94.4% and 98.3% respectively.

It can be observed that B cells and T cell populations are more closely related to each other than to monocytes. This is illustrated by the fewer regions of significant difference between their Raman spectra, the appearance of some overlap in the scatter plot clustering, and indeed the sensitivity and specificity achieved between pairwise comparisons. This data is as expected because CD4+ T cells and B cell originate from a common lymphoid progenitor lineage, whereas monocytes originate from a common myeloid progenitor lineage.

### Digital holographic microscopy

**Figure 2:**
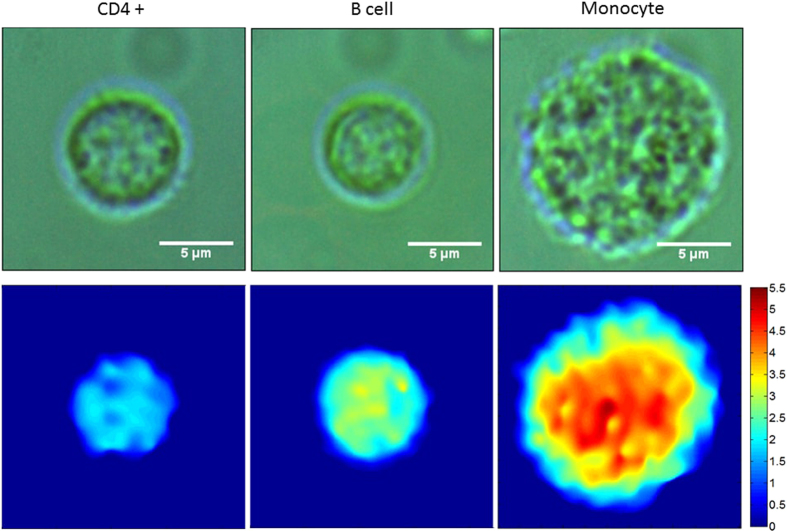
The top row shows example bright field images for each of CD4+ T cell, B cell, and monocyte cells (left to right). Scale bars denote 5 *μ*m in length. The bottom row represents respective phase images showing the phase difference between the signal and reference arm. The intensity is related to the optical path difference between the reference and signal arms which can be related to both absolute cell thickness and intracellular structure. Colour bar denotes the phase difference in the unit of radian.

**Figure 3:**
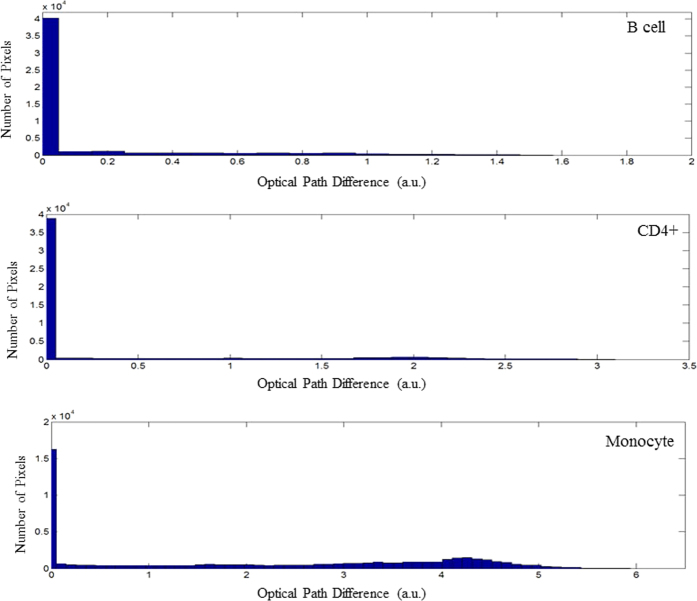
An example histogram for each of B cell, CD4+ T cell, and monocyte cell subsets. The histogram illustrates the number of pixels which relate to specific OPD values for a single cell phase map. Cells which induce a greater OPD between the signal the reference arm will have high OPD values, smaller cells will have a greater number of zero-value pixels. All phase map images are made up of 220 × 220 pixels.

**Figure 4:**
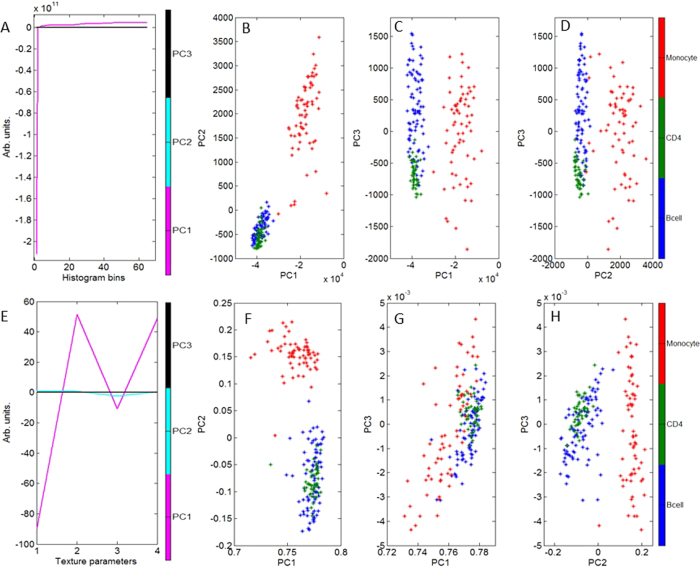
The top row (**A**–**D**) illustrates discrimination ability of DHM when a pixel intensity histogram is used to describe the phase map images. (**A**) Represents the PC loadings where the most significant contribution comes from number of zero intensity pixels indicating cell size is the biggest discriminating factor. (**B**–**D**) Represent PC scatter plots for all cell types using the first 3 PCs. The bottom row (**E**–**H**) illustrates discrimination ability of DHM when four texture parameters are used to describe the phase map images. Parameters 1–4 are contrast, correlation, energy and homogeneity respectively. (**F**–**H**) Represent the PC scatter plots for all cell types using the first 3 PCs. Each method forms a distinct cluster for monocytes indicating they are easy to identify using DHM, however B cells and CD4+ T cells appear to be more difficult to successfully differentiate. Using higher order PCs can see differences between the more closely related CD4+ and B cells as noticed in (**C**). Using a pixel intensity histogram appears to be more effective at discriminating between cell types than texture analysis.

Phase map images were generated for every cell. Example images for each cell type are illustrated in Fig. 2 alongside a respective bright field image. It can be seen from the bright field images that it is very difficult to distinguish between T cells and B cells morphologically, where they have a similar size and shape. DHM images provide a measure of the optical thickness of each cell, where phase differences are related to both the absolute thickness of the cell and its intracellular structure. We have found that mapping the optical path length difference across the cell volume was sufficient for discrimination purposes; specific knowledge of the absolute thickness and intracellular structure was not necessary. Conducting PCA directly on DHM images is computationally intensive and so the images were subsequently analysed in three different ways;

1. A histogram was created which summarised the number of pixels at specific optical path difference (OPD) values across the whole image. An example histogram for each cell type is illustrated in Fig. 3. These histograms can approximate information such as cell size: the number of non-zero pixels makes up the total area of the cell (or equally the more pixels with an intensity value of zero indicating a smaller cell area), maximum OPD: the largest pixel intensity value, and total OPD: the total of all non-zero elements. PCA could potentially also recognize other patterns in the histograms such as how evenly spread pixel values are, indicating uniformity across the phase image. Representing DHM images in terms of their pixel intensity histogram provided an array of values on which we could conduct PCA and use LOOCV statistics to discriminate between the cell subsets. Excellent discrimination efficiencies were achieved and are summarised in Table 2. An average sensitivity and specificity of 97.5% and 95.1% were achieved respectively. The PC scatter plots can be seen in Fig. 4B–D. The first PC is excellent at identifying monocytes, these are morphologically quite different from the other cell subsets, as they are larger and have a larger total OPD. Figure 4A shows the loadings for the first 3 PCs used in discrimination. PC1 indicates that most discrimination is based on cell size; this can be seen by the large contribution from the first histogram bin, which represents zero intensity pixels. This correlates well with what we see in Fig. 3. Higher order PCs can recognise more subtle differences between cell types; this is indicated in Fig. 4C where the third PC is better equipped to differentiate between B cells and CD4+ T cells. It can also be observed that B cells and monocytes show a greater morphological variation within their populations, compared to CD4+ T cells, as indicated by the spread of data points for monocytes and B cells, and the tightly formed cluster of CD4+ T cells.
2. Texture analysis was used to study the statistical properties of an image, resulting from the gray level co-occurrence matrix (GLCM). Here we have used the entire image as we are interested in differences between cell subsets as opposed to different regions within a cell. The four texture parameters chosen are contrast, correlation, energy and homogeneity. Table 3 represents the average values for each of these parameters for each cell, and their respective standard deviations. It can be observed that the most useful of these parameters is contrast which shows the most variation, particularly between monocytes and T cells or B cells. These four values formed a descriptor vector for each cell creating a new data set on which PCA was conducted and LOOCV was used to estimate the discrimination efficiencies. Pairwise sensitivities and specificities are summarised in Table 2, with an average sensitivity and specificity of 92.2% and 86.4% respectively. PCA scatter plots can be seen in Fig. 4F–H. The loadings for the first 3 PCs can be seen in Fig. 4E which indicates the most significant contribution to the first PC is from the first texture parameter ‘Contrast’. The second PC has its most significant contribution from the third texture parameter ‘Energy’, which plays an important role in differentiating monocytes from CD4+ T cells and B cells, as observed in Fig. 4F. It appears that texture analysis is not very effective at discriminating between CD4+ T cells and B cells, which correlates with the sensitivities and specificities found in Table 3. To expand this descriptor vector we investigated looking at texture parameters along four directions (0°, 45°, 90° and 135°), creating a 16 value vector, although this showed no improvement with respect to discrimination efficiencies. This is to be expected as there is no directional features across a whole cell, such as you may expect to see in tissue samples, furthermore the cells are imaged at random orientations on the slide.
3. The two methods described above can describe different features of the image. We therefore investigated combining the two descriptor vectors, where each cell is described by both 4 texture parameters and its signal intensity histogram. PCA and LOOCV were conducted on this combined-vector data set. It was found that the confusion matrix showed no improvement with respect to that achieved by only a histogram. We concluded that using solely a histogram to describe the phase map images was the most efficient method for analysis.

### Combining Raman spectroscopy and DHM

Raman spectra were used to discriminate between 3 cell subsets based on their respective chemical information and DHM images were used to discriminate between the 3 cell subsets based on their morphological profile. We investigated whether combining these complimentary signatures would provide a more complete analysis and improve discrimination abilities. To achieve this we added the Raman spectrum from each cell with the pixel-value intensity histogram calculated from its corresponding phase map to create a new descriptor vector for each cell. This was treated as a new dataset by PCA and LOOCV. The resulting sensitivities and specificities can be seen in Table 2. Interestingly the average sensitivity and specificity (92.2% and 96% respectively) for this combined, multi-modal analysis did not provide an improvement from discrimination based on individual techniques. Looking at individual pairwise sensitivities and specificities it can be observed that there is an improvement in discrimination efficiencies for B cell versus monocytes, and for CD4+ T cells versus monocytes, with respect to a pure Raman or DHM analysis, however the ability to discriminate between CD4 and B cells is weaker when compared to using solely Raman analysis. In conclusion we do not see that combining these data sets can always provide an improvement to discrimination ability.

### Flow cytometric characterisation of purified cell subsets

Immune cell subsets were isolated by negative depletion from human PBMC to obtain untouched primary cells. Flow cytometry of isolated cells from two separate donors revealed purity levels of 89% and 91% for CD4+ T cells, 80% and 91% for CD8+ T cells, 100% and 100% for B cells, and 96% and 99% for monocytes (Supplementary Data Fig. S1). This data therefore utilises freshly isolated human lymphocytes of direct biomedical relevance, in comparison to previous studies of established cultured cell lines^5^.

## Discussion

Using a multi-modal system has enabled the independent and simultaneous acquisition of both a Raman signature and quantitative phase information from key immune cell subsets. Each method has shown successful discrimination between all three cell types, of particular interest is between B cells and T cell subsets which are morphologically very similar, but perform very distinct functions in the immune system of secreting antibodies and cytokines, respectively.

RS can discriminate between the cells based on its molecular composition, main differences arise from DNA profile as well as protein and lipid content. We have further established RS ability to discriminate between CD8+ T cells, CD4+ T cells, B cells and monocytes in the supplementary material. One of the main challenges facing the implementation of Raman spectroscopy in a clinical system is the slow data acquisition times; for this reason we have combined RS with a faster modality as a step towards overcoming this limitation.

DHM was used to gain quantitative phase information on each cell type, providing a phase map image of each cell. The most effective way we found to describe these phase images was to form a histogram of pixel intensity values across a region of interest containing a single cell. Discrimination based on these histograms was achieved due to cell size, total OPD or phase volume (indicative of cell thickness and intracellular structure) and uniformity across the image. Texture analysis was successful at differentiating monocytes from CD4+ T cells and from B cells, but not very effective at discriminating between B cells and CD4+ T cells. The most dominant texture parameter for discrimination was contrast, which is a measure of the intensity contrast between a pixel and its neighbour across the whole image, giving an indication of the variance across an image. We have chosen to use laser irradiation for DHM measurements, a long coherence length offers the benefit of simple alignment and simple analysis, however it gives relatively low spatial coherence and is not able to separate refractive index and thickness values. For our discrimination purposes this simple set-up is sufficient; however it useful to note that other techniques could offer a more sophisticated analysis^31^ and may be implemented for future applications. This includes using light with short coherence length which can offer higher spatial resolution, such as diode lasers^32^ or even white light^33^,^34^. Using dual wavelength illumination^35^,^36^, scanning the illumination angle^37^,^38^, or introducing phase shifts^39^ can provide separable information on sample thickness and refractive index.

Possible physical interpretations for observed phase variations between B and T cells could be related to differences in amount of cytoplasm, stippled chromatin, or nuclear morphology, such as nuclear size, homogeneity, nuclear folds, thickness of nuclear membrane, or presence and uniformity of nucleoli^40^,^41^.

The combination of RS and DHM has several benefits which are summarised below:

1. The two modalities are able to validate each other for a more robust analysis.
2. DHM has a much faster acquisition time than RS, which may provide a fast initial screening where RS can focus on cells of interest for a more specific molecular analysis.
3. Potential biological applications may benefit from a system which can provide both chemical and quantitative phase information in parallel.

Interestingly we observed greater variation within the B cell subset, both in RS and DHM scatter plots. This could be attributed to variations within the cell type, such as memory or naive B cell subsets and would be interesting to investigate further.

Additionally each modality provides information not available from the other providing both a molecular and a morphological signature of each cell, providing an accurate discrimination of key immune cell subsets in a completely label-free manner.

## Methods

### Ethics Statement

This study was approved after ethical review by the School of Medicine Ethics Committee, University of St Andrews and all methods were carried out in accordance with guidelines and regulations. All samples were obtained with informed written consent.

### Cell purification

Blood samples were obtained from normal healthy donors after ethical review and with informed written consent obtained. 10 to 30 ml samples were collected into heparinised tubes and separated by centrifugation over Histopaque (Sigma, Poole UK) to obtain peripheral blood mononuclear cells (PBMC). Cells were washed in PBS containing 0.5% fetal calf serum (FCS, Life Technologies, Paisley UK). Immune cell subsets were isolated using Dynabead (Life Tehnologies) untouched human CD4+ T cell kit (containing depleting antibodies anti-CD8, CD14, CD16, CD19, CD36, CD56, CDw123 and CD235a), untouched human B cell kit (depleting antibodies anti-CD2, CD14, CD16, CD36, CD43 and CD235a) and untouched human monocytes (depleting antibodies anti-CD3, CD7, Cd16, CD19, CD56, CDw123 and CD235a).

### Flow cytometry

Cells were blocked in PBS plus 2% FCS supplemented with 20% human serum, then labelled with antibodies (eBiosciences, Hatfield UK), for CD4+ T cells (PE-CD14 or PE-CD4), for B cells (FITC-CD3 or FITC CD19) and for monocytes (FITC-CD3 or FITC-CD14). Flow cytometry was performed using a Guava 8HT (Millipore, UK) running Guavasoft 2.5.

### Raman set-up

The system was equipped with a Ti-Sapphire Laser (MSquared, UK, Solstis) with an incident wavelength of 785 nm used for standard Raman measurements. The light is focused through a 60x objective (Nikon 0.80 NA) providing 150 mW of power to the sample plane. The light is collected in reflection, through the same objective, and focused into a 200 *μ*m fibre. Raman photons are collected by a monochromator (Shamrock SR-303i, Andor Technology) with a 400 lines/mm grating, blazed at 850 nm, and a deep depletion, back illuminated and thermoelectrically cooled CCD camera (Newton, Andor Technology). Single point standard Raman data were taken with an acquisition time of 5 s per cell.

### DHM set-up

**Figure 5:**
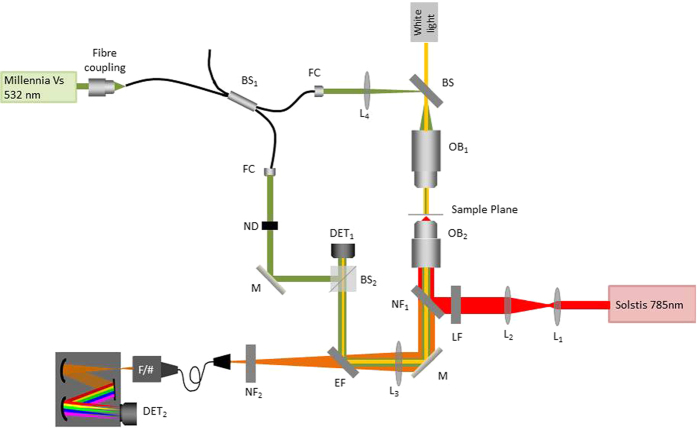
Schematic for multimodal system combining DHM and Raman spectroscopy. L_1_–L_4_: lens, M: Mirror, LF: laser line filter (Semrock, LL01-785-12.5), EF: edge filter (Semrock, Di02-R561), FC: fibre collimator (Thorlabs, F220FC_A), ND: neutral-density filter, OB_1_: Mitituyo M Plan Apo, Magnification = 10x, NA = 0.23, WD = 20 mm, OB_2_: Nikon CFI, Magnification = 60x, NA = 0.80, BS_1_: fibre beam splitter (Thorlabs, FC532_50B_FC), BS_2_: cube beam splitter, NF_1_: notch filter (Semrock, NFD01-785-25x36), NF_2_: notch filter (Semrock, NF03-785E), F/#: F-number matching optics, DET_1_: CMOS camera (Imaging Source, DFK 42AUC03), DET_2_: CCD camera (Andor Technology, Newton DU920P_DR).

An off-axis transmission DHM system was developed using a continuous wave diode-pumped solid-state laser (Spectra-Physics Millennia Vs, wavelength of 532 nm). The laser light was coupled into a single mode optical fibre splitter. The beam from one arm illuminated the sample along the same path as the bright field illumination and was focussed through a long working distance objective (Mitutoyo UK, M Plan Apo 10x 0.23NA). The beam passed through the sample, in a quartz chamber as described below, and was collected by a 60x objective (Nikon 0.85 NA). The two beams were recombined in a cube beam splitter at an angle to achieve an off-axis hologram on a complementary metal–oxide–semiconductor (CMOS) detector (Imaging Source, DFK 42AUC03, 1024 × 960, 8 bit, 25 fps). Images were taken with an exposure time of 10 ms. We reduced the power, by misalignment of the optical fibre coupling objective, to give a low power illumination (30 *μ*W) in the sample plane. The DHM illumination wavelength was chosen to be outside of the Raman emission range making it possible to combine the two optical systems and obtain both Raman and DHM data independently and simultaneously. It was not necessary to move the sample stage between the two methods of data collection for each cell. Figure 5 illustrates a schematic for this system. Due to the environmental requirements for a double path interferometer, such as low vibrations and temperature stability, our set-up was built on a floating table in a laboratory with temperature constantly monitored and controlled. A common path interferometer would be considered in the future to be less sensitive to such fluctuations and more robust for use outside of specialist laboratories.

### Sample preparation

A sample chamber was formed using a thick quartz slide (25.4 mm × 25.4 mm, 1 mm thickness, SPi Supplies, UK) and an 80 *μ*m vinyl spacer to form a well. 18 *μ*l of cell suspension in PBS was placed in the well and covered by a thin quartz slide (25.4 mm × 25.4 mm, 0.15 mm to 0.18 mm thick). The sample was allowed to settle on the thinner slide (for around 30 mins) before being placed on the microscope with the thin slide towards the objective. Letting the cells stick to the glass prevents any movement that may be caused by optical forces.

A total of 60 CD4+ T cells, 86 B cells and 67 monocytes were analysed using both standard RS and DHM. Previous studies have shown that there was no inter-donor variability amongst T cell populations, NK cells or dendritic cell populations^4^. There was no variability observed between data collected on separate days confirming the stability of the system. After being continuously exposed under 150 mW laser at 785 nm for 10 minutes, no changes in the Raman spectrum was observed. This confirmed that there is no laser damage to cells at this laser dosage. It is worth noting that incident laser light at 532 nm is collimated before the sample plane, ensuring a very low optical intensity is incident on the sample. No obvious damage was observed under bright light and no change to the Raman spectra was noted.

## Data Analysis

### Principal component analysis

PCA is well established for its use in RS but not many studies show its use with DHM. PCA is an orthogonal linear transformation of data to a new coordinate system in such a way as to reduce the dimensionality of the data set such that a small number of components (PC’s) may effectively describe the variance across the data set, where the first PC accounts for the greatest variance, the second PC accounts for the second greatest variance and so on. First the data is normalised by subtracting the mean and then the covariance matrix is calculated. From the covariance matrix we find the eigenvalues and respective eigenvectors. The eigenvectors represent the direction of the axis and the eigenvalue represents how much variance is in the data set. The eigenvector with the largest eigenvalue is therefore the first PC. The first few PCs can be selected as the new coordinate system and the normalised data is projected on the new coordinate system. More information can be found in ref. 42.

### Leave-one-out cross-validation

Discrimination efficiencies were calculated according to the method of leave-one-out cross-validation (LOOCV), in which the PCs are calculated from a data set with all but one data point^43^,^44^. This LOOCV spectrum is then projected onto the new space defined by the principal components and characterised according to the nearest neighbour algorithm. This is repeated for all the acquired data points. Correct and incorrect cell classifications are summarised in a confusion matrix. Sensitivity and specificity were then estimated for each pairwise cell subsets.

#### Raman spectroscopy

The Raman spectra are analysed in the region of 900 cm^−1^ to 1700 cm^−1^. The Raman spectra are initially normalised according to the total spectral intensity, to compensate for any power fluctuations. A student’s T-test was applied to compare cell lines in a pairwise manner; this highlighted spectral regions of significant difference indicating how two cell lines chemically differ from each other. The total data set from all cell lines was taken into account to form a training dataset. PCA was applied to this dataset to reduce dimensionality and the first 10 principal components (PCs) were selected. Discrimination efficiencies were calculated according to the method of leave-one-out cross-validation (LOOCV), which is repeated for all the acquired Raman spectra (see above). We performed an additional analysis using a third party background subtraction algorithm^45^ to validate our results (not shown here). We note that the sensitivities and specificities achieved with background subtraction did not change significantly compared to those presented here, demonstrating our data and method of analysis are robust.

#### DHM

The interference signal detected on the CMOS sensor contains both intensity and phase information; the off-axis configuration provides higher frequency interference fringes. This has the benefit of enhancing the spatial separation between coherent terms on the Fourier transform. The first coherent term (+1 diffraction term) can be separated from the zero-order and conjugate terms. Shifting this to the centre removes the carrier frequency. Taking an inverse Fourier transform of this then gives a wrapped phase map with low frequency fringes. Note if any Raman light was picked up by the detector it would not interfere with the reference beam and will be filtered out during this process. A second order quadratic fit was applied to compensate for any difference in wave-front curvature. Finally phase unwrapping resulted in a phase map of the cell. The use of an interferometric configuration can be very sensitive to vibrations; short acquisition times, such as ours, in the milliseconds range, can minimize the influence of vibrations on the image. For further optimisation we took a series of images for each individual cell and selected the image with the greatest contrast. PCA can not be conducted directly on the phase map images as this is computationally heavy. To characterise the images three methods were investigated;

1. A histogram representing the number of pixels in a specific signal intensity region was calculated. Information may be extracted from the histogram such as size of image, maximum optical path difference (OPD), and total OPD over the entire cell. Each histogram is treated as a descriptor vector for its respective cell. The total data set from all cells were considered to form a training set. PCA was conducted on this new data set, and each cell was classified according to LOOCV and the nearest neighbour algorithm.
2. Texture analysis is a statistical method quantifying certain parameters that can describe the properties of an image; such as smoothness, contrast, or regularity based on the relationship between neighbouring pixel intensities. As a first step a grey level co-occurrence matrix (GLCM) is calculated; this finds the frequency with which a pixel of grey scale intensity i occurs adjacent to a pixel of intensity j. Four texture parameters ‘Contrast’, ‘Correlation’, ‘Energy’, and ‘Homogeneity’ were then used to describe the entire image. Contrast is a measure of local variations in an image, correlation measures the frequency with which a pixel pair occurs across the image, energy is the sum of squared elements in the GLCM which can be thought of as the uniformity across the image, and finally homogeneity considers the amount of dominant grey-tone transitions in the image^25^,^26^,^27^. These four values formed a new descriptor vector for each cell, the total dataset was taken into consideration to form a training set, and PCA was conducted on this new data set in the same way as described above, using LOOCV and nearest neighbour algorithm to calculate sensitivity and specificity. It was also investigated if calculating the GLCM along four directions would provide a more complete description; 16 parameters could be calculated from the four texture parameters along 0°, 45°, 90° and 135° providing a 16-value descriptor vector.
3. The histogram and TA vectors described above were combined to form a new descriptor vector, capable of characterising size, OPD, and texture parameters. PCA was conducted on this combined histogram and TA dataset, using LOOCV and nearest neighbour methods to calculate discriminate efficiency.

## Additional Information

**How to cite this article:** McReynolds, N. *et al*. Multimodal discrimination of immune cells using a combination of Raman spectroscopy and digital holographic microscopy. *Sci. Rep.* **7**, 43631; doi: 10.1038/srep43631 (2017).

**Publisher's note:** Springer Nature remains neutral with regard to jurisdictional claims in published maps and institutional affiliations.

## Supplementary Material

Supporting Information

## Figures and Tables

**Table 1:** Confusion matrix illustrating the efficiency of RS to discriminate between cells.

Actual/Predicted	CD4+	B cells	Monocytes
CD4+	46	1	1
B cell	7	69	1
Monocyte	1	1	51

Values on the diagonal represent those correctly identified, off-diagonal values represent those incorrectly identified. There is most confusion between CD4+ and B cells as they are most closely related.

**Table 2:** Summary of sensitivities and specificities achieved for each technique.

	Raman Spectroscopy		DHM Histogram		DHM Texture analysis		Combined RS and DHM	
	sens %	spec %	sens %	spec %	sens %	spec %	sens %	spec %
CD4+ v B cell	86.8	98.6	93.8	85.4	78.0	62.2	81.3	88.3
CD4+ v monocytes	97.9	98.1	98.7	100	98.5	97.0	100	100
B cell v monocytes	98.6	98.1	100	100	100	100	98.6	100
**Average**	**94**.**4**	**98**.**3**	**97**.**5**	**95**.**1**	**92**.**2**	**86**.**4**	**93**.**3**	**96**.**1**

RS and DHM are each capable of efficiently discriminating between cell subsets. Describing phase images in terms of a pixel intensity histogram is more efficient than using texture parameters.

**Table 3:** Average Texture parameters for each cell subset.

	Contrast	Correlation	Energy	Homogeneity
CD4+	0.0388	0.9971	0.6718	0.9853
(std. dev.)	0.0091	0.0006	0.0294	0.0012
B cell	0.0382	0.9961	0.6619	0.9828
(std. dev.)	0.0082	0.0015	0.0637	0.0032
Monocyte	0.0803	0.9963	0.4121	0.9692
(std. dev.)	0.0161	0.0008	0.0347	0.0043

The most useful parameters for discrimination appear to be Contrast and Energy.
