# Supplementary material for: Multimodal discrimination of immune cells using a combination of Raman spectroscopy and digital holographic microscopy
Source: Sci Rep. 2017 Mar 3;7:43631. doi: 10.1038/srep43631 (PMC5335250; doi:10.1038/srep43631)
Supplement: Supporting Information [file srep43631-s1.pdf]

Supporting information for:
Multimodal discrimination of immune cells using a combination of Raman spectroscopy and digital holographic microscopy

Naomi McReynolds[1], Fiona G.M. Cooke[2], Mingzhou Chen[1], Simon J. Powis[2], and Kishan Dholakia[1,*]

[1]SUPA, School of Physics and Astronomy, University of St Andrews, Fife, KY16 9SS, United Kingdom
[2]School of Medicine, University of St Andrews, Fife, KY16 9TF, United Kingdom
[*]kd1@st-andrews.ac.uk

Supplementary Information

Figure S1 illustrates the purity levels of isolated cell subsets as analysed by Fluorescence activated cell sorting (FACS).

In addition to the main manuscript we use this section to demonstrate the validity of Raman spectroscopy to discriminate between further immune cell subsets. Firstly we expand our data set to include four immune cell subsets: CD8+ T cells, CD4+ T cells, B cells and monocytes. A total of 57 CD4+ T cells, 175 CD8+ T cells, 52 B cells, and 127 monocytes were analysed over 2 days from a single donor. The first 10 PCs were used which accounts for 98.7% of the total variance across the data set. Table S1 shows the confusion matrix achieved and table S2 illustrates pairwise sensitivities and specificities achieved. Figure S2 illustrates the Raman spectra achieved for each cell type and the scatter plots achieved using the first three PCs. Scatter plots for the four cell types show distinct clusters illustrating their ability to be identified. As noted in the main manuscript we observe a large spread of B cell data points, which could be attributed to variations within the cell type, such as memory or naive B cell subsets. It is important to highlight that these results were obtained on a different Raman system indicating this technique is valid across different systems. All parameters were kept constant: an incident wavelength of 785 nm was used and 150 mW power was provided in the sample plane. RS were acquired over 5 s and the spectra were analysed in the same manner over the spectral region 900 cm^{-1} to 1700 cm^{-1}.

Actual/ Predicted	CD4	CD8	B cells	Monocytes
CD4	56	1	0	0
CD8	0	169	4	2
B cell	0	13	39	0
Monocyte	0	4	1	122

Table S1. Confusion matrix for cells characterised by RS. Values on the diagonal represent those correctly identified, off-diagonal values represent those incorrectly identified

	Sensitivity %	Specificity %
CD4 V CD8	100	99.4
CD4 V B cell	100	100
CD4 V Monocyte	100	100
CD8 V B cell	92.9	90.7
CD8 V Monocyte	97.7	98.4
B cell V Monocyte	97.5	100

Table S2. Pairwise sensitivities and specificities achieved for immune cell subsets analysed by Raman spectroscopy

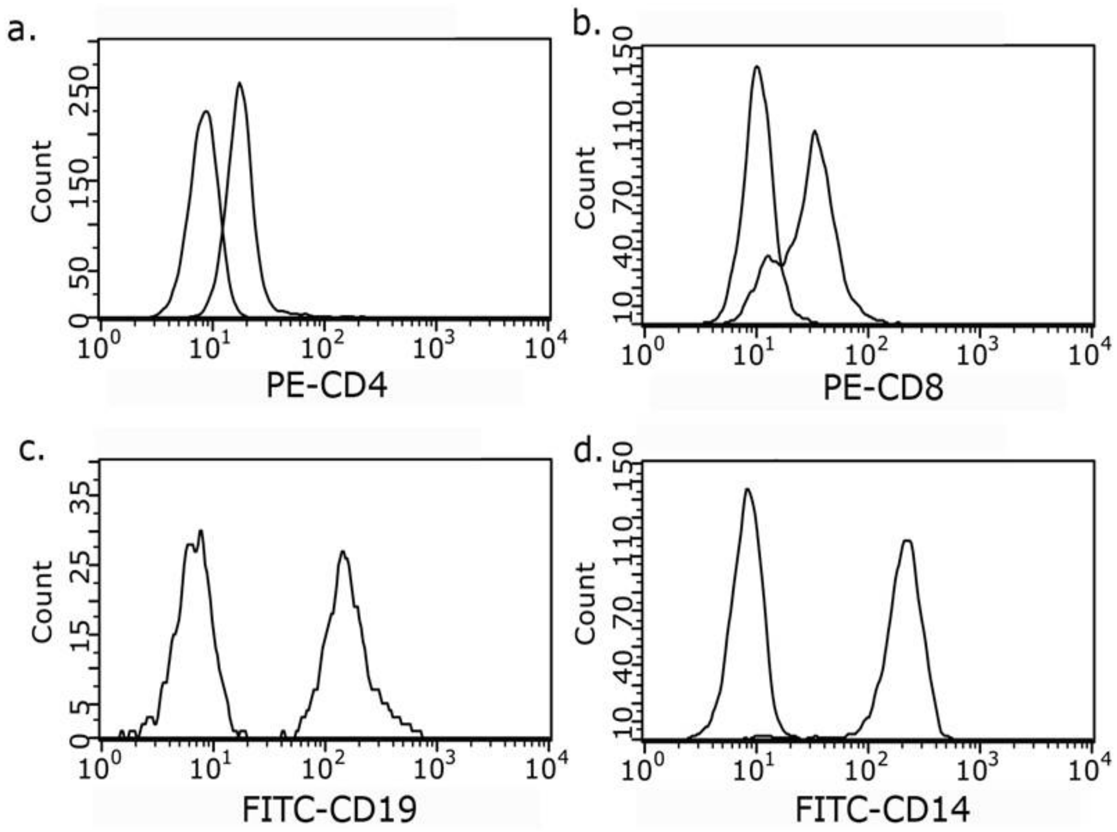

Figure S1. Facs analysis of a) CD4 + T cells, b) CD8 + T cells, c) B cells, and d) monocytes from two donors revealing purity levels of 89% and 91% for CD4+ T cells, 80% and 91% for CD+ T cells, 100% and 100% for B cells, and 96% and 99% for monocytes. In all panels the left hand curves indicate staining with an irrelevant antibody, as listed in the methods section.

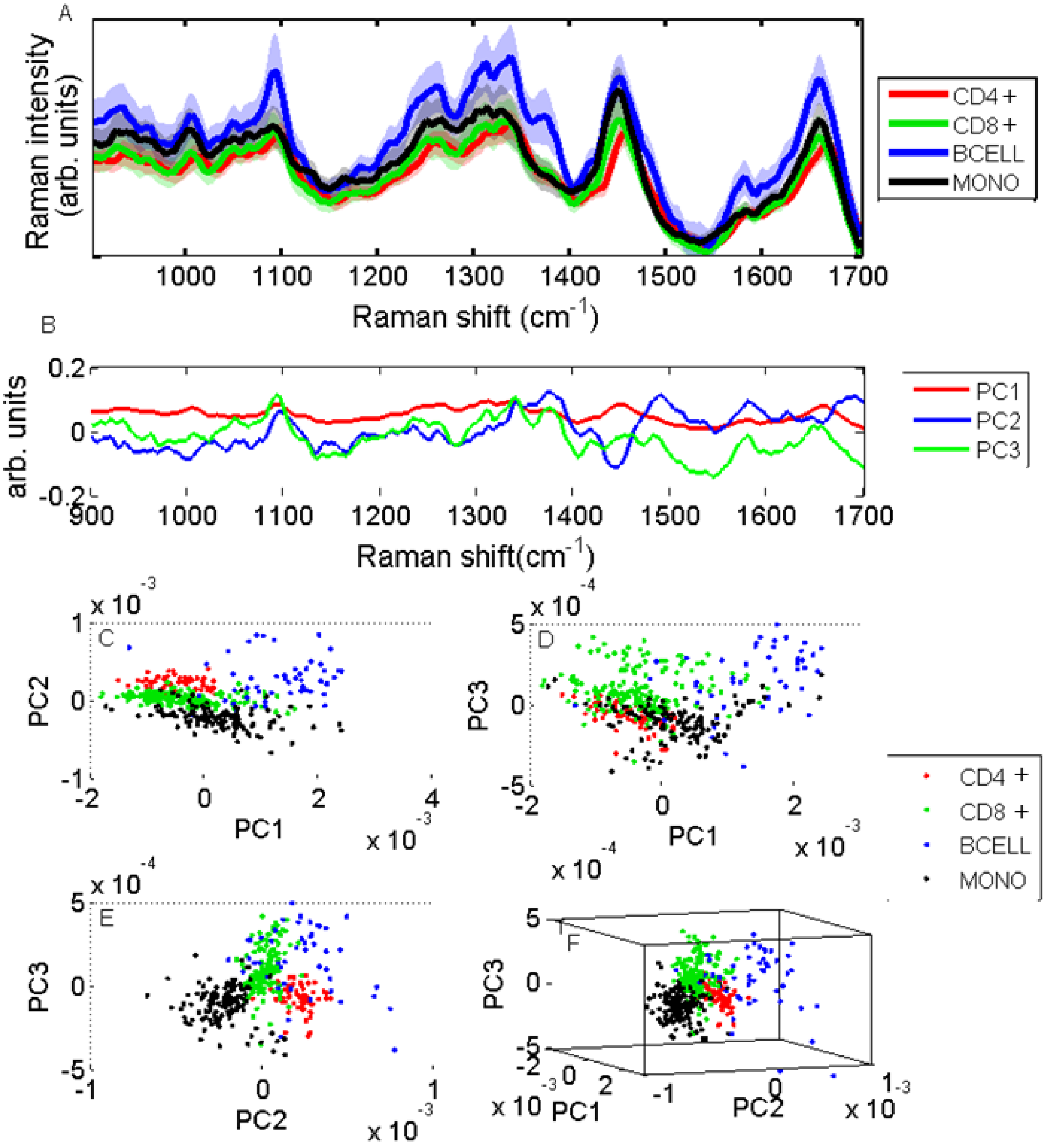

Figure S2. A) illustrates the mean standard Raman spectrum for each cell type, where shadowed regions indicate the standard deviation. B) represents the loadings for the first three principal components for all cell types. C-F demonstrate scatter plots achieved using the first three PCs. The formation of distinct clusters indicates the ability of RS to successfully discriminate between cell types.
